# Individual and community level factors associated with anemia among lactating mothers in Ethiopia using data from Ethiopian demographic and health survey, 2016; a multilevel analysis

**DOI:** 10.1186/s12889-020-08934-9

**Published:** 2020-05-24

**Authors:** Alemneh Mekuriaw Liyew, Achamyeleh Birhanu Teshale

**Affiliations:** Department of Epidemiology and Biostatistics, Institute of Public Health, College of Medicine and Health Sciences, University of Gondar, Gondar, Ethiopia

**Keywords:** Anemia, Lactation, Multilevel analysis, Ethiopia

## Abstract

**Background:**

Maternal anemia is a worldwide public health problem especially in developing countries including Ethiopia. The anemia burden among lactating mothers was higher in Africa particularly in Ethiopia, and scant attention was paid. To date, there is limited evidence on community level determinants of anemia among lactating mothers in Ethiopia. This study, therefore, aimed to assess the prevalence and factors associated with anemia among lactating mothers in Ethiopia.

**Methods:**

Secondary data analysis was employed using the 2016 Ethiopian Demographic and Health Survey. A total weighted sample of 4658 lactating women was included. A multilevel logistic regression model was used to identify individual and community level determinants of anemia during lactation. Finally, the adjusted odds ratio with a 95% confidence interval was reported.

**Results:**

The overall prevalence of anemia was 28.3% (95% CI; 26.7, 30.0) with the higher regional prevalence in Somali (68.3%) and Afar (47.2%) regions. Current modern contraceptive use [AOR = 0.71; 95% CI: 0.58, 0.87], Poorer [AOR = 0.77; 95% CI: 0.61, 0.98], middle [AOR = 0.74; 95% CI: 0.56, 0.97], rich [AOR = 0.64; 95% CI: 0.46, 0.85], and richest [AOR = 0.66; 95% CI: 0.43, 0.98] wealth index, being working within the 12 months preceding the survey [AOR = 0.77; 95% CI: 0.64, 0.92], and taking iron during pregnancy [AOR = 0.82; 95% CI: 0.68, 0.98] were associated with lower odds of anemia. Whereas, being female household head [AOR = 1.22; 95% CI: 1.01, 1.49], having two births [AOR = 1.27; 95% CI: 1.04, 1.55] and three to four births [AOR = 1.53; 95% CI: 1.14, 2.06] within 5 years, and higher community illiteracy level [AOR = 1.06; 95% CI: 1.06, 1.70] were associated with the increased odds of anemia during lactation.

**Conclusion:**

In this study the prevalence of anemia among lactating mothers was high. It was affected by both individual and community level factors. Therefore, focusing on family planning services especially on modern contraceptive methods, iron supplementation during pregnancy, child spacing, and improving community literacy could decrease anemia during lactation.

## Background

Anemia refers to low hemoglobin in blood with a cutoff point < 110 g/L for pregnant women and < 120 g/L for non-pregnant women [1]. It is a condition which is characterized by a decreased number of red blood cells or hemoglobin level that results in insufficient oxygen-carrying capacity of blood to meet the cellular metabolic demand of the body. It is a worldwide public health problem affecting both developing and developed countries and all population groups [2]. The World Health Organization (WHO) defines anemia as a major public health problem, moderate public health problem, and mild public health problem when prevalence is over 40%, between 20 and 40%, and between 5 and 20% respectively [3].

Globally, 38% of pregnant women and 29% of non-pregnant women are anemic. Pregnant women in low-income and middle-income countries had high rates of anemia, in which the highest prevalence rates are reported in Central and West Africa (56%), South Asia (52%), and East Africa (36%) [4]. Iron deficiency is the major contributor to anemia globally and it is a major nutritional problem that accounted for 50% of the cases of anemia. However, folic acid, vitamin B12, and vitamin A deficiency can also cause nutritional deficiency anemia. In addition, acute and chronic inflammations, parasitic infections, and acquired or inherited disorders that affect the synthesis of hemoglobin and production or survival of red blood cells can be causes for anemia [2, 3].

Anemia in lactating mother is common especially if the mother were anemic during their pregnancy [5]. Lactating mothers are vulnerable to anemia morbidity due to their susceptibility to iron depletion during pregnancy and lactation as well as due to bad consequences of blood loss during their childbirth [6, 7]. Prevalence of anemia in lactating mother is different across different regions of the world; in Vietnam 66%, in India 63%, in Myanmar 60.3%, in Kenya 43.8%, and in china which is 32.7% [8–12]. In Ethiopia, the burden of anemia in lactating mothers ranges from 10.9 to 28.7% [13–15].

Anemia in lactating mother has various adverse effects like decreased immunity which in turn results in; delayed wound healing, and increased susceptibility to infections such as mastitis, ductitis, and urinary tract infection and diminished quality or volume of the breast milk. It has also associated with reduction of global household income, cognitive impairment, impaired quality of life, and emotional instability as well as postpartum depression [16–19]. These devastating impacts make anemia in lactating mothers to be one of the global health priority areas at the global level, especially in resource-limited areas [20].

Evidences revealed that different factors such as; maternal age, educational status of the mother, parity, wealth status, sex of household head, maternal body mass index (BMI), antenatal care (ANC) visit, cesarean delivery, history of a terminated pregnancy, smoking, health insurance, maternal occupation, religion, marital status, source of drinking water, type of toilet facility, place of delivery, iron supplementation during pregnancy, current modern contraceptive use, duration of breastfeeding, number of births within the past 5 years, birth interval, region, and place of residence are associated with anemia among lactating mothers [11, 15, 21–25].

Reducing anemia in women of reproductive age is considered an essential part of improving the health of a woman, and WHO has set a global target of achieving a 50% reduction of anemia among women of reproductive age by 2025 [26]. To achieve this target tackling anemia during lactation have its great importance. So far there are limited studies conducted in Africa particularly in Ethiopia and community level factors that might affect anemia during lactation were largely overlooked. Therefore, this study was aimed to assess the prevalence and the individual and community level factors associated with anemia during lactation. Since this is based on nationally representative data, it will give an insight for health professionals and policymakers in understanding the burden of anemia in lactating mothers and its determinants for setting possible interventions at both individual and community levels.

## Methods

### Study setting

The study was conducted in Ethiopia (3o -14o N and 33o - 48°E) which is located at the horn of Africa. The country covers 1.1 million Sq. km and has a great geographical diversity, which ranges 4550 m above sea level down to the Afar depression to 110 m below sea level. There are nine regional states and two city administrations subdivided into 68 zones, 817 districts, and 16,253 kebeles (lowest administrative units of the country) in the administrative structure of the country [27].

### Data source and sampling procedure

For this study, we used the 2016 Ethiopian Demographic and Health Survey (EDHS) data which was conducted from January 18, 2016, to June 27, 2016. It is a nationally representative data containing key health indicators such as; child and maternal health, and anemia among reproductive-age women. Besides, the sociodemographic and socioeconomic characteristics of the respondents were also collected and found in the survey.

**Fig. 1:**
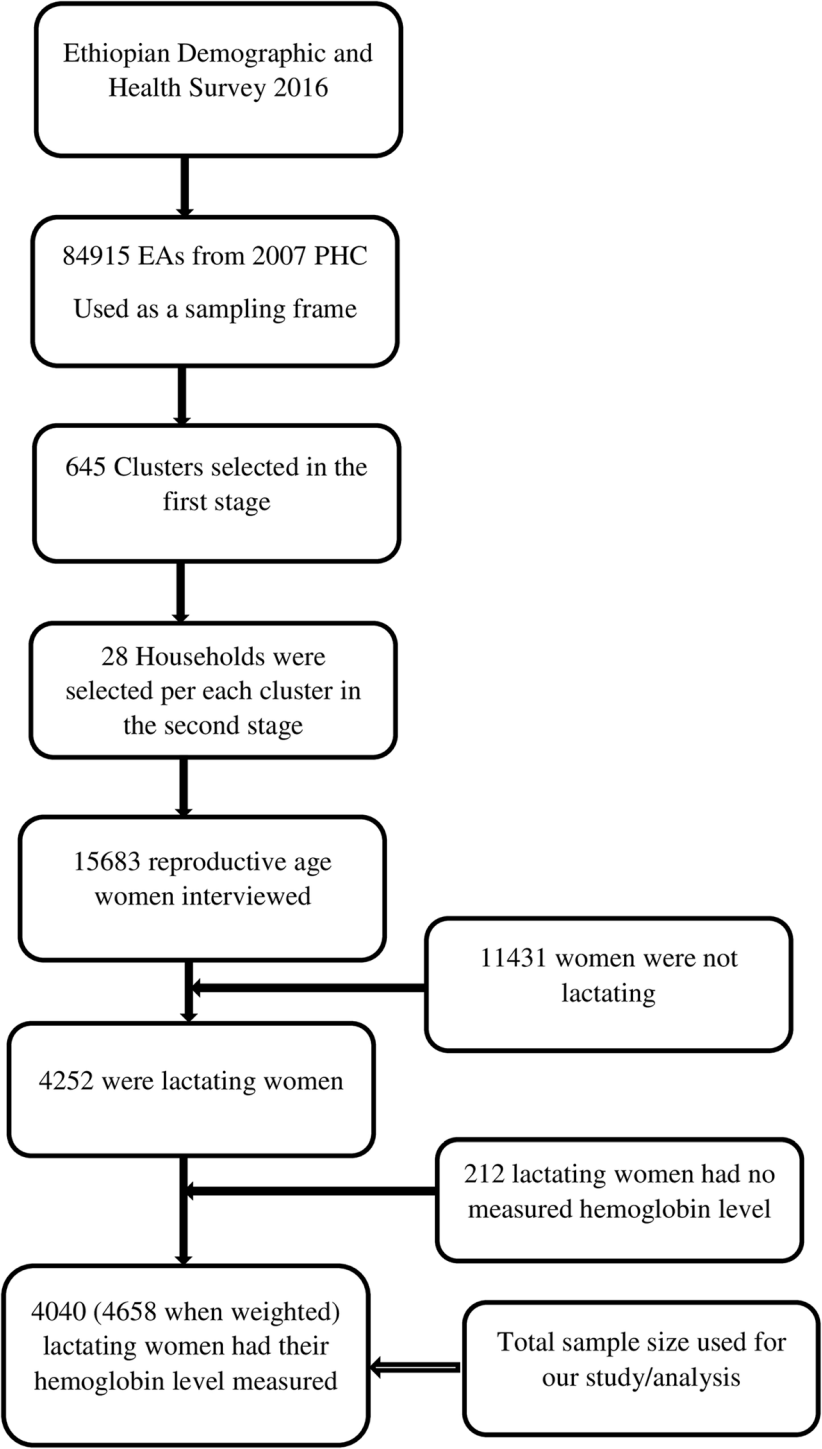
The flowchart showing the data extraction and sampling procedure

A stratified two-stage cluster sampling procedure was employed to select study participants. In the first stage, 645 enumeration areas (EAs) (202 urban and 443 rural) were selected from a list of 84,915 EAs created for the 2007 Ethiopia Population and Housing Census. Then, in the second stage, 28 households per each cluster were selected. A total of 16,583 eligible women were interviewed. The hemoglobin level was measured for those eligible reproductive-aged women and adjusted for altitude using the adjustment formula (adjust = − 0.032*alt + 0.022*alt2 and adjHb = Hb - adjust (for adjust > 0) [27, 28]. In the current study, a total weighted sample of 4658 lactating mothers was included. To reach such sample size several exclusion criteria were employed (Fig. 1). Further information regarding the data collection procedure, questionnaire, in general about how the survey was conducted, is found in the EDHS 2016 report [27].

### Variables of the study

#### Dependent variable

The current study was based on the altitude adjusted hemoglobin level which was already provided in the EDHS data. The outcome variable is anemia in lactating mother, which is operationalized as a categorical variable by predefined cut-off points for mild, moderate, and severe anemia recommended by the WHO for women above the age of 15 years. For this analysis, we recategorized anemia level as anemic and non-anemic from prior classifications in levels (no, mild, moderate, severe) because of very small numbers of cases in the categories of severe and mild anemia. Therefore, women with hemoglobin level < 120 g/L were considered as anemic and coded as “1” whereas those nonanemic were coded as “0” for our analysis.

#### Independent variables

For this study both individual and community level variables were considered. The mothers’ age, educational level of the mother, parity, wealth status, sex of household head, BMI, ANC visit, cesarean delivery, history of a terminated pregnancy, smoking, health insurance, maternal occupation, religion, marital status, perception of distance from the health facility, source of drinking water, type of toilet facility, place of delivery, iron supplementation during pregnancy, current modern contraceptive use, duration of breastfeeding, births within the past 5 years and birth interval were considered as individual level variables. Whereas community poverty, community media exposure, community illiteracy level, region, and place of residence were considered as community level variables. Some individual level variables were recategorized to make them suitable for analysis. The aggregate community level explanatory variables were constructed by aggregating individual level characteristics at the community (cluster) level. They were dichotomized as high or low based on the distribution of the proportion values computed for each community, after checking their distribution. Since all aggregated variable was not normally distributed, the median value was used as a cut-off point for categorization. Community poverty level was categorized as high if the proportion of women from the two lowest wealth quintiles in a given community was 50–100% and low if the proportion was 0–49%. Community media exposure was categorized as low if the proportion of women exposed to media in the community was 0–28.60% and categorized as high if the proportion above 28.60%. Community illiteracy level was categorized as high if the proportion of illiterate women per cluster was 83.3–100% and low if it was less than 83.30%. These independent variables (both individual and community level variables) were constructed/adapted from the searching of different literatures.

### Model building

Four models were fitted. The first was the null model (Model I) containing no exposure variables which was used to check the variability of anemia in the community and provide evidence to assess random effect using the intraclass correlation coefficient (ICC). The second (Model II) and the third (Model III) multilevel models contain individual level variables and community level variables respectively. In the fourth model (Model IV) both individual and community level variables were fitted simultaneously with the outcome variable. Model comparison was done using deviance and the model with the lowest deviance was selected as the best-fitted model.

### Parameter estimation method

The fixed effects (a measure of association) were used to estimate the association between the likelihood of anemia and explanatory variables at both community and individual levels and were expressed as odds ratios with 95% confidence interval. Regarding the measures of variation (random-effects), median odds ratio (MOR), ICC, and Proportional Change in Variance (PCV) were estimated.

The MOR aims to translate the area level variance in the widely used odds ratio scale, which has a consistent and intuitive interpretation. The MOR is defined as the median value of the odds ratio between the area at the highest risk and the area at the lowest risk when randomly picking out two areas or clusters. The MOR can be conceptualized as the increased risk (in median) that would have if moving to another area with a higher risk.

It is computed by; MOR = exp.[√(2 × Va) × 0.6745], where; VA is the area level variance, and 0.6745 is the 75th centile of the cumulative distribution function of the normal distribution with mean zero and variance one [29, 30]. Whereas the PCV reveal the variation in anemia among lactating mother across communities which is explained by both individual and community level factors. The PCV is calculated as; PCV = [(VA-VB)/VA]*100 [30, 31], where; VA = variance of the initial model, and VB = variance of the model with more terms. Moreover, the ICC which reveals the variation of anemia between clusters is calculated as; ICC = [VA/(VB + 3.29)], where; VA = area/cluster level variance, and VB = individual level variance [30].

## Results

### Sociodemographic characteristics of study participants

In this study, we used weighted samples of 4658 breastfeeding/lactating women. The median age of the study participants was 28 (IQR = 24–33) years. About half (49.52%) of study subjects had no formal education and the majority (39.17%) of respondents were followers of orthodox religion. Regarding household status, 22.64 and 22.63% of study subjects were from the poorest and poorer households. The majority (74.70%) of lactating mothers had normal nutritional status (BMI = 18.5–24.99) and 95.21% of respondents were not covered by health insurance. Most (72.29%) of respondents had no media exposure and 59.29% perceived distance from the health facility as a big problem. Looking to source of drinking water and type of toilet facility, 42.97 and 91.21% of study subjects used unimproved water sources for drinking and unimproved toilet facility respectively. Regarding respondent’s residence and region, 89.26 and 96.65% of respondents were from rural residence and agrarian regions respectively (Table 1).

**Table 1:** Sociodemographic characteristics of lactating mothers in Ethiopia, 2016

Variables	Frequency	Percentage
Maternal age		
15–19	249	5.32
20–29	2306	49.52
30–39	1733	37.20
40–49	370	7.90
Maternal education		
No formal education	2916	62.61
Primary education	1395	29.95
Secondary education	247	5.30
Higher education	100	2.14
Religion		
Orthodox	1824	39.17
Protestant	1017	21.82
Muslim	1673	35.91
Other*	144	3.10
Current marital status		
Married	4383	94.11
Unmarried	275	5.89
Maternal occupation		
Working	1196	25.67
Not working	3462	74.33
Wealth status		
Poorest	1055	22.64
Poorer	1054	22.63
Middle	1017	21.85
Rich	868	18.63
Richest	664	14.25
Sex of household head		
Female	606	13.00
Male	4052	87.00
Maternal BMI		
< 18.5	943	20.25
18.5–24.99	3479	74.70
≥ 25	263	5.05
Smoking		
Yes	32	0.68
No	4626	99.32
Health insurance coverage		
Yes	224	479
No	4434	95.21
Perception of distance from the health facility		
Big problem	2762	59.29
Not big problem	1896	40.71
Media exposure		
Yes	1291	27.71
No	3367	72.29
Source of drinking water		
Pipe	1242	26.66
Other improved	1415	30.37
Not improved	2001	42.97
Type of toilet facility		
Improved	405	8.69
Not improved	4253	91.31
Place of delivery		
Home	3104	66.65
Health facility	1554	33.35
Delivery by CS		
Yes	115	2.46
No	4543	97.54
History of a terminated pregnancy		
Yes	382	8.19
No	4276	91.81
ANC visit		
No visit	1592	34.18
one-three	1513	32.48
four and above	1553	33.34
Iron supplementation during pregnancy		
No	2482	53.28
Yes	2176	46.72
Current modern contraceptive use		
Yes	1615	65.34
No	3043	34.66
Duration of breastfeeding		
≤ 12 months	2242	48.13
13 to 36	1483	31.84
37 and above	933	20.03
Births in the five years		
One	2461	52.84
Two	1854	39.80
Three to four	343	7.37
Parity		
Primiparous	919	19.71
Multiparous	1935	41.53
Grand multiparous	1804	38.75
Birth interval		
< 24 month	579	15.47
≥ 24 month	3159	84.53
Residence		
Urban	501	10.74
Rural	4157	89.26
Community media exposure		
Low	2130	45.72
High	2528	54.28
Community illiteracy level		
Low	2444	52.48
High	2214	47.52
Community poverty level		
Low	2818	60.49
High	1839	39.51

Other* = catholic, traditional & other

### Prevalence of anemia among lactating mothers in Ethiopia, 2016

The prevalence of anemia in this study was 28.3% (95% CI; 26.7, 30.0). Regarding the regional prevalence of anemia during lactation, the highest prevalence was observed in the Somali region (68.3%) followed by the Afar region (47.2%).

### Random effect and model comparison

Table 2 revealed the random effect or community variation and model comparison/fitness. As indicated from the table, the ICC in the null model was 0.21, which means about 21% of the variations of anemia in lactating mothers were attributable to the difference at cluster level or community level factors. The higher MOR value (2.46) in the null model also revealed that anemia among lactating mothers was different between clusters or EAs. Furthermore, the higher PCV value (0.44) in the final model indicates that about 44% of the variation of anemia among lactating mothers was attributable to both the individual level and community level factors. Regarding model comparison/fitness, we used deviance and the model with the lowest deviance value (Model IV) was the best-fitted model.

**Table 2:** Random effect and model comparison for factors associated with anemia among lactating mothers in Ethiopia, 2016

Parameter	model I	Model II	Model III	Model IV
ICC	0.21(0.17–0.26)	0.13(0.10–0.18)	0.16(0.13–0.21)	0.13(0.10–0.18)
PCV	Reference	0.43	0.27	0.44
MOR	2.46(2.18–2.77)	1.97(1.75–2.20)	2.15(1.91–2.41)	1.95(1.75–2.20)
Model fitness				
Deviance	4895.418	4702.346	4787.795	4690.300

### Determinants of anemia among lactating mothers

In the bivariable multilevel logistic regression analysis all factors, except maternal age, smoking, marital status, birth interval, health insurance coverage, and religion, were associated with anemia in lactating mother (*p* < 0.20). In the multivariable analysis household wealth status, maternal working status within the 12 months preceding the survey, sex of household head, current modern contraceptive use, iron supplementation during their last pregnancy, number of births within 5 years and community illiteracy level was significantly associated with anemia in lactating women (*p* < 0.05).

Mothers from poorer, middle, rich and richest households had 23% [Adjusted odds ratio (AOR) = 0.77; 95% CI: 0.61, 0.98], 26% [AOR = 0.74; 95% CI: 0.56, 0.97], 36% [AOR = 0.64; 95% CI: 0.46, 0.85], and 34% [AOR = 0.66; 95% CI: 0.43, 0.98] lower odds of having anemia as compared to mothers from poorest households. Looking at the sex of the household head, being mothers from households with female household head had 1.22 [AOR = 1.22; 95% CI: 1.01, 1.49] times higher odds of having anemia. The odds of anemia were 23% [AOR = 0.77; 95% CI: 0.64, 0.92] lower among lactating mothers who had been working within the 12 months preceding the survey as compared to their counterparts. The odd of having anemia was 18% [AOR = 0.82; 95% CI: 0.68, 0.98] lower in mothers who took iron during their last pregnancy as compared to their counterpart. Lactating mothers who use modern contraceptive methods currently have 29% [AOR = 0.71; 95% CI: 0.58, 0.87] lower odds of having anemia. Regarding the number of births within 5 years, lactating mothers who had two and three to four births had 1.27 [AOR = 1.27; 95% CI: 1.04, 1.55] and 1.53 [AOR = 1.53; 95% CI: 1.14, 2.06] times higher odds of anemia as compared to mothers who had one birth within 5 years. Moreover, lactating mothers from communities with higher illiteracy had 1.34 [AOR = 1.06; 95% CI: 1.06, 1.70] times higher odds of anemia as compared to their counterparts (Table 3).

**Table 3:** A multivariable multilevel analysis of factors associated with anemia among lactating mothers in Ethiopia, 2016

Variables	Model I	Model II AOR (95%CI)	Model III AOR (95%CI)	Model IV AOR (95%CI)
Maternal education				
No formal education		1.00		1.00
Primary education		0.96(0.79–1.17)		1.00(0.82–1.22)
Secondary education		1.07(0.76–1.52)		1.14(0.80–1.63)
Higher education		1.41(0.85–2.35)		1.51(0.90–2.52)
Maternal occupation				
Working		0.76(0.63–0.91)		0.77(0.64–0.92) **
Not working		1.00		1.00
Wealth status				
Poorest		1.00		1.00
Poorer		0.71(0.57–0.89)		0.77(0.61–0.98) *
Middle		0.64(0.50–0.83)		0.74(0.56–0.97) *
Rich		0.53(0.40–0.70)		0.64(0.46–0.85) *
Richest		0.52(0.37–0.74)		0.66(0.43–0.98) *
Sex of household head				
Female		1.23(1.02–1.50)		1.22(1.01–1.49) *
Male		1.00		
Maternal BMI				
18.5–24.99		1.00		1.00
< 18.5		1.2(1.00–1.43)		1.78(0.99–1.41)
≥ 25		0.84(0.61–1.15)		0.82(0.60–1.13)
Perception of distance from the health facility				
Big problem		1.00		1.00
Not big problem		0.97(0.82–1.15)		1.00(0.84–1.18)
Media exposure				
Yes		0.91(0.75–1.11)		0.92(0.75–1.14)
No		1.00		1.00
Source of drinking water				
Pipe		1.00		1.00
Other improved		1.18(0.93–1.50)		1.18(.93–1.51)
Not improved		1.14(0.90–1.45)		1.13(0.89–1.45)
Type of toilet facility				
Improved		1.00		1.00
Not improved		0.80(0.61–1.04)		0.82(0.62–1.07)
Place of delivery				
Home		1.00		1.00
Health facility		0.93(0.76–1.14)		0.96(0.78–1.18)
Delivery by CS				
Yes		0.81(0.49–1.34)		0.81(0.49–1.34)
No		1.00		
History of a terminated pregnancy				
Yes		0.76(0.57–1.02)		0.76(0.57–1.02)
No		1.00		1.00
ANC visit				
No visit		1.00		1.00
one-three		1.05(0.85–1.30)		1.06(0.86–1.32)
four and above		0.97(0.77–1.23)		0.99(0.78–1.25)
Iron supplementation during pregnancy				
Yes		0.81(0.68–0.97)		0.82(0.68–0.98) *
No		1.00		1.00
Current modern contraceptive use				
Yes		0.69(0.56–0.84)		0.71(0.58–0 .87) ***
No		1.00		1.00
Duration of breastfeeding				
≤ 12 months		1.00		1.00
13 to 36		0.88(0.74–1.05)		0.89(0.74–1.06)
37 and above		0.89(0.70–1.12)		0.90(0.71–1.14)
Births in the five years				
One		1.00		1.00
Two		1.29(1.06–1.57)		1.27(1.04–1.55) *
Three to four		1.59(1.19–2.14)		1.53(1.14–2.06) **
Parity				
Primiparous		1.00		1.00
Multiparous		1.07(0.83–1.37)		1.08(0.84–1.39)
Grand multiparous		1.21(0.92–1.60)		1.23(0.94–1.62)
Residence				
Urban			1.00	1.00
Rural			1.38(0.99–1.91)	1.13(0.75–1.72)
Community media exposure				
Low			1.00	1.00
High			0.91(0.73–1.13)	0.96(0.77–1.20)
Community illiteracy level				
Low			1.00	1.00
High			1.60(1.26–2.04)	1.34(1.06–1.70) *
Community poverty level				
Low			1.00	1.00
High			1.65(1.28–2.12)	1.15(0.88–1.51)

*AOR* Adjusted Odds Ratio, *CI* Confidence Interval, * = *P* < 0.05, ** = *P* < 0.01 and *** = *P* ≤ 0.001

## Discussion

Anemia in lactating mother is a neglected public health problem which has its impact for both the mother and the newborn [12]. Thus, we investigated the prevalence and determinants of anemia among lactating women in Ethiopia.

In this study, the prevalence of anemia among lactating mothers was 28.3% and this was in line with a study done in India [32] and Ethiopia [14]. The prevalence of anemia in lactating mothers found in this study was lower than studies conducted in China, India, Vietnam, and Myanmar [9–12]. This might be because mothers near to and after giving birth are granted sufficient maternity leave according to the social and cultural norms in Ethiopia, which also allows them to get adequate rest. Also, in Ethiopia, lactating mothers are allowed to eat a range of foods like animal products, even during the fasting period. It may also be because Teff Injera (which has a higher iron content) is the staple Ethiopian food eaten by the majority of the country’s population [33].

However, the current finding is higher than a previous study in Ethiopia using EDHS 2011 data [15]. This might be attributed to increased coffee, tea, and red wine consumption (both alcoholic and non-alcoholic) from time to time, which decreases iron absorption and induces anemia [34, 35]. This high prevalence of anemia in lactating mothers can result various adverse consequences both for the mother and the child mostly due to its impact in the immune system; such as maternal death, clogged milk ducts, mastitis, thrush, and diminished quality or volume of the milk [19].

Consistent with a study in Ethiopia based on EDHS 2011 [15], we found that lactating mothers who had been working within the 12 months preceding the survey had lower odds of having anemia. This might be because mothers who were working can have a good income and buy the necessary and variety of foods including iron-containing foods [36]. The other plausible explanation is that mothers who worked would have a great deal of trust and decision-making ability to have sufficient feeding practice.

Similarly, mothers who were from poorer, middle, rich, and richest households had lower odds of having anemia as compared to those from poorest households. This finding is supported by studies in Nepal [24], Myanmar [11], Rwanda [23], and Ethiopia [15]. This might be because mothers from rich households had a great opportunity to have a balanced diet in terms of meal frequency and variety of food [37].

This study also indicated that mothers who had supplemented with iron during their last pregnancy had lower odds of having anemia as compared to their counterparts. This is supported by a study in Bahir Dar-Ethiopia [38], which showed that iron supplementation during pregnancy is negatively associated with having anemia both for pregnant and lactating women. The possible explanation could be, iron is the most important nutrient which is used for the formation of red blood cells and when it was taken during pregnancy it can have a probability of preventing anemia during the locational period as well.

Consistent with a study done in Nepal [24] and Ethiopia [21], which indicated being male household head lower the chances of the mothers to be anemic, in our study households who had female head had higher odds of anemia as compared with those households whose head was male. This may be due to the fact that awareness towards anemia and treatment-seeking behaviors for any health problems might be lower in female-headed households [21]. This study also revealed that lactating mothers who were taking modern contraceptives had a lower risk of having anemia. This is congruent with studies done in low and middle-income countries [22], sub-Saharan Africa [39], Rwanda [23], and Ethiopia [15]. This is because taking contraceptives could potentially minimize the monthly (menstrual) bleeding [40, 41]. Moreover, by reducing a woman’s likelihood of becoming pregnant, oral contraceptives may also reduce the risk of anemia from antepartum or postpartum hemorrhage.

The number of births a woman had with 5 years is another factor associated with anemia among lactating mothers which revealed that mothers who had two and three to four children within 5 years had higher odds of having anemia. This finding is supported by a study in Ethiopia [42] which showed having too frequent birth is among a significant predictor of anemia. This is since too many births in a short period (within 5 years) might not give enough time to replenish or substitute lost nutrient stores before another reproductive cycle starts and result in iron deficiency anemia [43]. Besides, mothers with frequent birth might have both antepartum and postpartum hemorrhage with consecutive births, which in turn result in chronic and repeated anemia.

Moreover, in our study higher community illiteracy level was another important factor which was associated with higher odds of having anemia in lactating mother. Another study also revealed that maternal health service utilization is associated with literacy level in the community in which mothers from communities with higher illiteracy level had higher odds of utilizing maternal health services [44]. The possible reason for the association of women illiteracy level with anemia in lactating mothers might be lower education level decreases communication within the family particularly with the husband on health-related issues. In addition, illiteracy prevents women from developing the confidence to make decisions regarding their health and seek out quality health services [45, 46]. In remote societies, higher proportions of illiterate females also indicate lower autonomy which in turn results in restriction from accessing important maternal health care services during pregnancy, childbirth, and the postpartum period which finally end up with comorbidities like anemia.

This study had both limitations and strengths. Among limitations, some important variables which are known to cause anemia such as helminthic infection and protozoan infections like malaria were not assessed. In addition, due to the cross-sectional nature of the data, it is difficult to show a cause and effect relationship between independent and dependent variables. The main strength of this study was the use of nationally representative data with a large sample size, based on laboratory-confirmed anemia, which makes the findings of the study more representative to all lactating mothers in Ethiopia. The other strength is since it is based on the national survey it has the potential to give an insight for policy-makers and program planners to design intervention strategies at the individual and community level.

## Conclusion

In this study the prevalence of anemia among lactating mothers was high. Both individual and community level factors were associated with anemia among lactating mothers. Mothers from rich households, those who had been working in the 12 months preceding the survey, current modern contraceptive users, those mothers who had been taking iron during the last pregnancy, having more than one number of births within 5 years had lower odds of having anemia. While being mothers from households with female household heads and being from communities with higher proportions of illiterate women increases the odds of having anemia. Therefore, focusing on family planning services especially on modern contraceptive methods, iron supplementation during every pregnancy, child spacing, and improving community literacy could decrease anemia during lactation.

## Data Availability

All relevant data are available within the manuscript.

## Abbreviation

ANC: Antenatal Care
AOR: Adjusted Odds Ratio
BMI: Body Mass Index
EAs: Enumeration Areas
EDHS: Ethiopia Demographic and Health Survey
MOR: Median Odds Ratio
PCV: Proportional Change in Variance
SNNPR: Southern Nation Nationality and People Region
ICC: Intraclass Correlation Coefficient
WHO: World Health Organization
